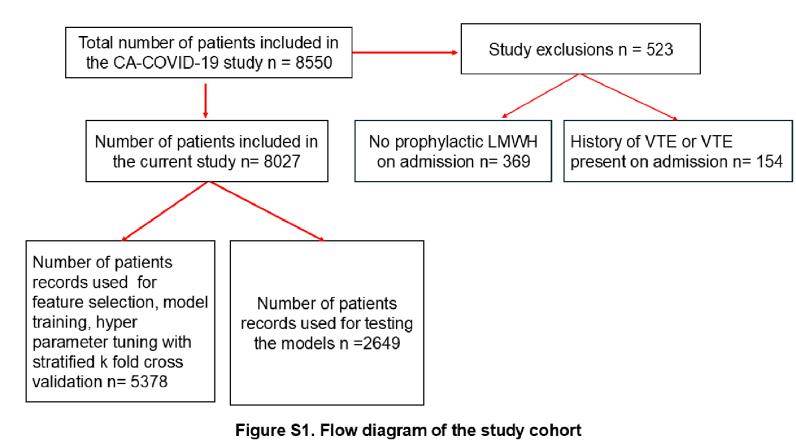# Comparison of 7 artificial intelligence models in predicting venous thromboembolism in COVID-19 patients

**DOI:** 10.1016/j.rpth.2025.102711

**Published:** 2025-02-27

**Authors:** Indika Rajakaruna, Mohammad Hossein Amirhosseini, Mike Makris, Mike Laffan, Yang Li, Deepa J. Arachchillage

**Affiliations:** 1 Department of Computer Science and Digital Technologies, University of East London, London, United Kingdom; 2 Department of Cardiovascular Sciences University of Sheffield, Sheffield, United Kingdom; 3 Department of Immunology and Inflammation, Centre for Haematology, Imperial College London, London, United Kingdom; 4 Department of Haematology, Imperial College Healthcare NHS Trust, London, United Kingdom

**Keywords:** artificial intelligence, COVID-19, deep learning, machine learning, thrombosis

## Abstract

**Background:**

An artificial intelligence (AI) approach can be used to predict venous thromboembolism (VTE).

**Objectives:**

To compare different AI models in predicting VTE using data from patients with COVID-19.

**Methods:**

We used feature ranking through recursive feature elimination with AI algorithms (logistic regression and random forest classifier) and standard statistical methods to identify the significant factors that contribute to developing VTE in COVID-19 patients using a large dataset from “Coagulopathy associated with COVID-19,” a multicenter observational study. We developed 7 AI models (Multilayer perceptron classifier, Artificial neural network with backpropagation, eXtreme gradient boosting, Support vector classifier, Stochastic gradient descent classifier, Random forest classifier and Logistic regression classifier) using the selected significant features to predict the development of VTE during hospitalization and used K-fold cross-validation and hyperparameter tuning to validate and optimize the models. The models’ predictive power was tested on 2649 (33% of 8027 overall patients), which were previously separated and not used during model training and validation stages.

**Results:**

Age, female sex, white ethnicity, comorbidities (diabetes, liver disease, autoimmune disease), and laboratory features (increased hemoglobin, white cell count, D-dimer, lactate dehydrogenase, ferritin), and presence of multiorgan failure were major factors associated with the development of thrombosis. Support vector classifier (SVC) model outperformed all other models, achieving an accuracy of 97%. The SVC model also led in precision (0.98), recall (0.97), and F1 score (0.97), and recorded the lowest log-loss score (0.112 on the test dataset), reflecting better model convergence and an improved fit to the data. Additionally, it achieved the highest area under the curve score (0.983).

**Conclusion:**

The SVC model delivered the best overall performance outperforming similar studies that developed deep learning and machine-learning models for COVID-19.

## Essentials

• VTE is a major complication in COVID-19 patients.
• An AI approach can be used to predict VTE.
• Seven AI models using the selected significant features were developed to predict VTE.
• SVC model outperformed all other models, achieving an accuracy of 97%.

## 1 Introduction

Artificial intelligence (AI) in medical diagnosis and treatment has revolutionized the field of healthcare, offering new levels of accuracy and efficiency. AI technologies, particularly in medical diagnostics, are transforming how diseases are detected, analyzed, and treated. By utilizing machine-learning and deep-learning algorithms, AI can process a large amount of data quickly and accurately, providing healthcare providers with invaluable insights [1]. Key areas in medicine that use AI are development of disease or clinical outcome prediction models and tracking the spread of disease or infections. It played a significant role in COVID-19, which was first declared as a global pandemic in early 2020 [2,3]. Thrombosis was a major complication in patients with COVID-19 admitted to hospitals. The risk of venous thrombosis in COVID-19 patients was 3 to 6 times higher than those hospitalized for other reasons [3].

Broadly, AI has been applied to COVID-19 in 4 key areas: diagnosis, public health, clinical decision-making, and therapeutics [4]. Several AI-driven studies have been published on predicting thrombosis in non–COVID-19 patients [5]. During and after the COVID-19 pandemic, there has been a surge in studies integrating AI to predict clinical outcomes in various contexts. Zhang et al. [6] developed an enhanced machine-learning model to improve deep venous thrombosis (DVT) prediction in COVID-19 patients. Their approach resulted in the creation of a DVT prediction model called bSES-AC-RUN-FKNN, which combines fuzzy k-nearest neighbor (FKNN) with an improved Runge-Kutta optimizer (RUN). The model incorporates key features such as age, sex, body mass index (BMI), prothrombin time (PT), international normalized ratio (INR), thrombin time (TT), C-reactive protein (CRP), tumor necrosis factor (TNF), interferon (IFN), and the neutrophil-to-lymphocyte ratio. The model achieved an accuracy of 91.02% and a sensitivity of 91.07%. However, its performance was constrained by the limited dataset size, including only 424 patients, of whom 202 developed DVT [6].

AI will play a significant role in the future of prediction models and clinical practice, as it has the ability to enhance precision, efficiency, and scalability in clinical medicine, facilitating better outcomes for the patients. Using AI prediction models to improve patient outcome has the potential to reduce costs and prepare for the future, making medicine more proactive, accessible, and effective.

Selecting the best model is the key in AI as the model’s performance directly impacts patient safety and clinical outcomes in addition to ethical considerations. Selection of the best AI prediction model significantly benefits biostatisticians and researchers by enhancing research accuracy, facilitating precision medicine, efficient data analysis, increasing the prediction power, and preparing for future challenges effectively. However, it must be mentioned that there is an important ongoing debate on the use of AI methodology as its implementation raises some issues related to safety, ethical accountability, and how effectively it can be integrated into clinical practice. Good quality, reliable data representing a diverse population are fundamental in developing successful AI models that can be applicable to the general population because poor quality or biased datasets lead to inaccurate and unsafe predictions. Strict regulation of privacy and data security, especially when data are shared across institutions or between countries, is vital as breach of data security is a major ethical concern. Effective integration of AI into clinical practice requires infrastructure, training for clinicians to understand the systems, and interdisciplinary collaboration.

Although COVID-19 is no longer a major threat to public health due to mass vaccination and antiviral treatment of patients who develop severe infection, models developed using data obtained from patients with COVID-19 can be used as examples for other disease conditions that may share some common risk factors. Although COVID-19 disease was itself a major cause of venous thromboembolism (VTE), some patient risk factors are common to many diseases that are associated with VTE.

In this study, using a large set of data obtained from a multicenter observational study conducted across 26 UK National Health Service (NHS) Trusts (Coagulopathy in COVID19-A Multi-Centre Observational Study in UK https://www.clinicaltrials.gov/ct2/show/NCT04405232), we aimed to compare the ability of different AI models to predict VTE in hospitalized COVID-19 patients to obtain the best-performing model. Data from the Coagulopathy in COVID-19 study have been used to assess the clinical outcomes such as thrombosis, major bleeding, multiorgan failure (MOF), and mortality, as well as their associations with patient demographics, comorbidities, and admission laboratory data, using standard statistical methods [[7], [8], [9], [10], [11], [12]].

## 2 Methods

### 2.1 Data source

The study was approved by the Health Research Authority (HRA), Health and Care Research Wales, and received local Caldicott Guardian approval in Scotland (reference number: 20/HRA/1785). The dataset comprises records of 8027 COVID-19 patients aged 18 years and older admitted to hospitals between April 1, 2020, and July 31, 2020. VTE was defined as radiologically confirmed pulmonary embolism and/or DVT. All patients received prophylactic dose low molecular weight heparin on admission to the hospital unless contraindicated, for example, by the presence of a bleeding disorder or platelet count < 30 × 10^9^/L. However, all patients included into this study received prophylactic dose low molecular weight heparin. Patients who had VTE at admission were excluded (Supplementary Figure S1) and only patients with symptomatic VTE, who had imaging (Doppler scans or CT pulmonary angiogram or CT scans) to confirm VTE during hospitalization, were considered to have VTE.

### 2.2 Data cleansing and feature engineering

During the data preprocessing stage, we identified outliers and invalid data using scatter plots, data sorting, and IQR calculations. We also applied constraints to certain features, such as human weight, height, and blood test results, to ensure no unrealistic values were recorded and k-Nearest Neighbors imputation strategy was used to account for missing laboratory values (<10%) of D-dimer, troponin I, ferritin, and lactate levels but not for comorbidities or clinical outcomes. Once the imputation was done, results were reviewed for each imputed feature to make sure that the imputation had generated plausible data.

Body mass index (BMI) and age were categorized using clinically relevant cutoff points (“<18.5,” “18.6-24.9,” “25-29.9,” “30-39.9,” “>40”) and (“18-29 years,” “30-49 years,” “50-69 years,” “70-89 years,” “>90 years”), respectively. A full list of features is given in Table 1.

**Table 1:** Features used to identify the significant factors for the model development.

Demographics features	Comorbidities	Laboratory results
Sex (male/female)	Multiorgan failure	Hemoglobin
Ethnicity (White/Asian/Black)	History of smoking	Platelets
Age (y)	History of liver disease	D-dimer
Age groups (y); (18-29, 30-49, 50-69, 70-89, >90)	History of lung disease	White cell count
BMI groups (kg/m^2^) (0-18.5, 18.6-24.9, 25-29.9, 30-39.9, >40)	History of diabetes	Neutrophils
	History of heart disease	Lymphocytes
	History of hypercholesterolemia	Fibrinogen
	History of hypertension	Alanine transferase
	History of malignancy	Bilirubin
	History of autoimmune disease	Creatinine
	History of bleeding disorder	C-reactive protein
		Lactate dehydrogenase
		Troponin I
		Ferritin
		Prothrombin time
		Activated partial thromboplastin time
		Lactate

Categorical clinical and demographic features were encoded using a one-hot encoding scheme, which created binary columns for each category. Numerical features, such as laboratory test results, were standardized using a standard scaler to bring the values within a consistent range (normalization). This was done to prevent varying feature scales from biasing the model’s predictions, which could otherwise lead to higher misclassification errors and reduced accuracy.

### 2.3 Feature selection

Following initial feature selection based on clinical expertise, we employed multiple methods including (1) Statistical tests (t-test/Mann–Whitney U-test/chi-squared test); (2) Pearson pairwise correlation; (3) feature ranking through recursive feature elimination with logistic regression; and (4) random forest classifiers, to identify the features most relevant to thrombosis development in COVID-19 patients. All the features included in the study (demographics, comorbidities, laboratory features) are presented in Table 2.

**Table 2:** Demographics, clinical characteristics, and laboratory features of the 8027 COVID patients included in the study.

		Total *n* = 8027	Percentage
Sex	Male	4403	55%
	Female	3624	45%
Age (y)	18-29	207	3%
	30-49	991	12%
	50-69	2237	28%
	70-89	3864	48%
	>90	728	9%
Ethnicity	White	5811	72%
	Black	313	4%
	Asian	428	5%
	Other	1475	19%
Body mass index (kg/m^2^)	<18.5	215	3%
	18.6-24.	979	12%
	25.0-29.	5596	69%
	30-39.9	1007	13%
	>40.0	230	3%
History of liver disease	Yes	295	4%
	No	7732	96%
History of lung disease	Yes	1964	24%
	No	6063	76%
History of diabetes	Yes	2256	28%
	No	5771	72%
History of heart disease	Yes	1837	23%
	No	6190	77%
History of hypercholesterolemia	Yes	1265	16%
	No	6762	84%
History of hypertension	Yes	3740	47%
	No	4287	53%
History of malignancy	Yes	873	11%
	No	7154	89%
History of autoimmune disease	Yes	604	8%
	No	7423	92%
History of bleeding disorders	Yes	59	1%
	No	7968	99%

**Laboratory features**			
**Laboratory results**	**Median**	**Inter quartile**	**Reference range**
Hemoglobin (g/L)	130	114-143	130-160 (115-150)^a^
	110^a^	98-134^a^	
Platelets (10^9^/L)	220	168-289	150-400
D-dimer (ng/mL)	1077	585-2851	0-500
White cell count (10^9^ /L)	7.68	5.5-7.8	4.1-11.1
Neutrophils (10^9^/L)	5.89	3.9-8.8	2.1-6.7
Lymphocytes (10^9^/L)	0.9	0.6-1.3	1.3-3.7
Fibrinogen (g/L)	5.6	4.3-6.8	1.5-4.5
Alanine transferase (IU/L)	26	17-43	8-40
Bilirubin (μmol/L)	10	7-14	0-20

a Female hemoglobin.

The t-test or Mann–Whitney U-test was used to compare groups based on the distribution of the data. Pearson correlation measures the strength of the linear relationship between 2 variables, with Pearson’s correlation coefficient quantifying this relationship for each feature with respect to the target label. In feature selection, pairwise correlation helps identify groups of highly correlated features, allowing the model to retain maximum predictive power while minimizing the number of features used. This approach enhances model’s efficiency and reduces redundancy. Recursive feature elimination (RFE) is an iterative process that fits a model, then removes the least important feature at each step until a specified number of features remain. RFE is a wrapper-type feature selection method because it relies on a machine-learning algorithm at its core to rank and select features. This distinguishes it from filter-based methods, which independently score each feature and select those with the highest or lowest scores.

We applied logistic regression and random forest classifier algorithms with RFE. RFE starts by including all features from the training dataset, then iteratively removes the least important features until the desired number remains. This is done by fitting the chosen machine-learning algorithm, ranking the features by importance, discarding the least important ones, and refitting the model. This process continues until the optimal subset of features is achieved.

Based on the common features identified through the feature selection methods (Table 3) and existing literature on COVID-19, we selected key features for the model training. These features included the presence of MOF; history of diabetes, liver disease, and autoimmune disease; age, female sex, white ethnicity; and levels of hemoglobin, white cell count, D-dimer, lactate dehydrogenase (LDH), and ferritin at hospital admission.

**Table 3:** Significant features for developing thrombosis clinical outcome of COVID-19 patients.

Feature selection method	Significant features
Statistical tests (t-test/Mann–Whitney U-test/chi-squared test)	Multiorgan failure (*P* < .001), White ethnicity (*P* < .014), history of diabetes (*P* < .019), history of autoimmune disease (*P* < .038), hemoglobin (*P* < .041), white cell count (*P* < .044)
Pearson pairwise feature correlation	Multiorgan failure, major bleeding, White ethnicity, history of diabetes, history of autoimmune disease, hemoglobin, white cell count, age (y)
Feature ranking with recursive feature elimination (logistic regression)	Multiorgan failure, major bleeding, Black ethnicity, age (y), history of liver disease, White ethnicity, history of autoimmune disease, history of diabetes, sex female
Feature ranking with recursive feature elimination (random forest regressor)	Raised levels of D-dimer, lactate dehydrogenase, ferritin, white cell count, creatinine, activated partial thromboplastin time

### 2.4 AI models

We developed and compared 7 binary classification AI models in predicting the risk of developing VTE in COVID-19 patients.

#### 2.4.1 Multilayer perceptron classifier

This artificial neural network (ANN) model employs a feedforward architecture, linking input data to corresponding output values through multiple interconnected layers. Each layer is connected to the next, with neurons utilizing nonlinear activation functions, except in the input layer. The network may contain 1 or more nonlinear hidden layers positioned between the input and output layers to enhance learning and predictive capabilities.

#### 2.4.2 ANN with backpropagation

Backpropagation is a process used to update the weights and biases of a neural network by calculating the difference between the predicted output and the true output. The algorithm propagates this error backward through the network, from the output layer to the input layer, adjusting the weights and biases of each neuron in the process to minimize the overall error and improve the model’s performance.

#### 2.4.3 eXtreme gradient boosting

It is a boosting algorithm utilizing bagging, where multiple decision trees are trained independently, and their results are combined to improve overall performance.

#### 2.4.4 Support vector classifier

It is an implementation of the Support Vector Machine algorithm and identifies the optimal hyperplane that maximally separates data points into distinct classes.

#### 2.4.5 Stochastic gradient descent classifier

This linear classification algorithm identifies the optimal decision boundary (hyperplane) to separate data points into different classes within a feature space. It works by iteratively adjusting the model’s parameters to minimize a cost function using the stochastic gradient descent (SGD) optimization technique.

#### 2.4.6 Random forest classifier

This method generates multiple decision trees using random subsets of both the data and features. Each decision tree acts as an independent "expert," offering its classification of the data. Predictions are made by aggregating the outputs of all trees and selecting the most frequent (or popular) result as the final prediction.

#### 2.4.7 Logistic regression classifier

This algorithm estimates the probability of an input belonging to a specific class. Although it is a (generalized) linear method, it applies the logistic function to transform predictions, ensuring the output is a probability value between 0 and 1.

### 2.5 Cross-validation and hyperparameter tuning

We applied 5-fold cross-validation (K = 5) to evaluate model performance, ensuring each train/test split was large enough to be statistically representative of the entire dataset. In K-fold cross-validation, the data is divided into K equal subsets (folds). The model is trained K times, each time using K-1 folds for training and the remaining fold for testing. The results from each iteration are averaged to provide a comprehensive assessment of model performance. This method helps to reduce the risk of overfitting and gives a more reliable indication of how the model will generalize to unseen data.

Grid Search and Random Search were also utilized for hyperparameter tuning to identify the optimal parameters for each model, maximizing their predictive power. Tuning these hyperparameters was critical, as they significantly impacted model performance. After evaluating various combinations using the 5378 records out of 8027, stratified K-fold cross-validation in the hyperparameter tuning resulted in the optimum parameters for getting the best possible performance for each model (Table 4).

**Table 4:** Optimum values for each model parameter after performing hyperparameter tuning.

Model	Modified hyperparameters and extra details
Multilayer perceptron classifier	Activation = relu, Hidden layer sizes = (100, 1), Learning rate = constant
Artificial neural network	Keras sequential API with optimizer function = adam, number of times to run the model = 10 and compilation with binary cross entropy loss function and rectified linear unit and sigmoid activation functions
eXtreme gradient boosting	Learning rate = 0.01, maximum tree depth = 3, and minimum child weight = 1
Support vector classifier	Strength of the regularization (C) = 3.4067, gamma = 0.331, probability estimates = True
Stochastic gradient descent classifier	Elastic Net mixing parameter (l1_ratio) = 0.05 loss = log, penalty (regularization term) = elasticnet
Random forest	Minimum number of samples at a leaf node = 5, Maximum depth of the tree = 4, function to measure the quality of a split = entropy
Logistic regression	Inverse of regularization strength (C) = 10

### 2.6 Performance measurements

The validity and predictive power of the models were evaluated using a confusion matrix and key performance metrics such as accuracy, precision, recall, F1 score, area under the receiver operating characteristic curve (ROC AUC), and log loss.

Confusion matrix provides a summary of prediction results by comparing actual vs predicted values, allowing for the calculation of important metrics like accuracy, precision, recall, and F1 score.

ROC AUC measures the model’s ability to distinguish between positive and negative cases, with a higher area under the curve (AUC) indicating better classification performance.

Precision is the proportion of predicted positive cases that are actually positive.

Recall is the proportion of actual positive cases correctly identified by the model.

F1 score is the harmonic mean of precision and recall, offering a single measure of performance by balancing both metrics.

Log loss is a performance metric used in classification problems, which reflects how close predicted probabilities are to actual values in binary classification, penalizing inaccurate predictions with higher values.

These metrics provide a comprehensive understanding of the model’s overall performance and predictive accuracy.

Hyperparameter tuning was used with ROC AUC scoring as a metric to get best hyper parameters for the model and then used ROC curve functionality to derive an optimum threshold for each model. When establishing the threshold, experts’ advice was taken on the threshold value matching for the binary classification problem that we try to resolve in these models (VTE is likely or not). Considering all these factors, we finalized the probability threshold at 0.4, which is an arbitrary threshold used for all the models. It is important to highlight that the arbitrary threshold will vary based on sensitivity and specificity of the prediction of VTE desired and what is the acceptable balance between risk of missing a VTE vs risk of causing bleeding by giving thromboprophylaxis

## 3 Results

Out of 8027 patients included in the study; 335 patients developed VTE (4.17%). We present the performance results of 7 AI models, which were built using a dataset of 5378 patient records. Testing was conducted with the remaining 2649 patient records. The data split for training and testing was done through a stratified shuffle split to prevent the imbalance in the distribution of classes in the training and testing datasets. As outlined earlier, the model development process included 5-fold cross-validation and hyperparameter tuning.

Among the models, the support vector classifier (SVC) demonstrated the best performance in predicting thrombosis, achieving an accuracy of 97%. It was 100% accurate in predicting the absence of VTE (true negatives) and outperformed other models in correctly predicting thrombosis development (true positives). Additionally, the SVC model excelled in key metrics, with a precision of 0.98, recall of 0.97, F1 score of 0.97, and the lowest log-loss score of 0.112 on the test dataset, indicating superior model convergence and data fitting. It also achieved the highest ROC AUC score of 0.983, highlighting its excellent ability to distinguish between positive and negative cases.

Following the SVC, the multilayer perceptron (MLP) classifier and ANN models showed a strong overall performance across metrics such as accuracy, precision, recall, F1 score and ROC AUC score, surpassing the other models in comparison. The MLP classifier and random forest models followed the SVC model closely with a loss score of 0.157, demonstrating reasonable convergence and a relatively low level of error during training. The MLP classifier followed the SVC model with an ROC AUC score of 0.685, demonstrating a good, though not as strong ability to differentiate between classes. Random forest and eXtreme gradient boosting (XGBoost) models also showed reasonably good ROC AUC scores (0.669 and 0.663 respectively). However, based on both the loss and ROC AUC scores as well, the SVC model stands out as the top performer, showing strong convergence during training and exceptional discriminatory power. The multilayer perceptron classifier, random forest, and SGD classifier followed the SVC model closely with accuracy scores of 0.96 however among them, multilayer perceptron classifier outperformed random forest and SGD classifier when evaluated using F1 score, precision, and recall metrics with a value of 0.94.

**Figure 1:**
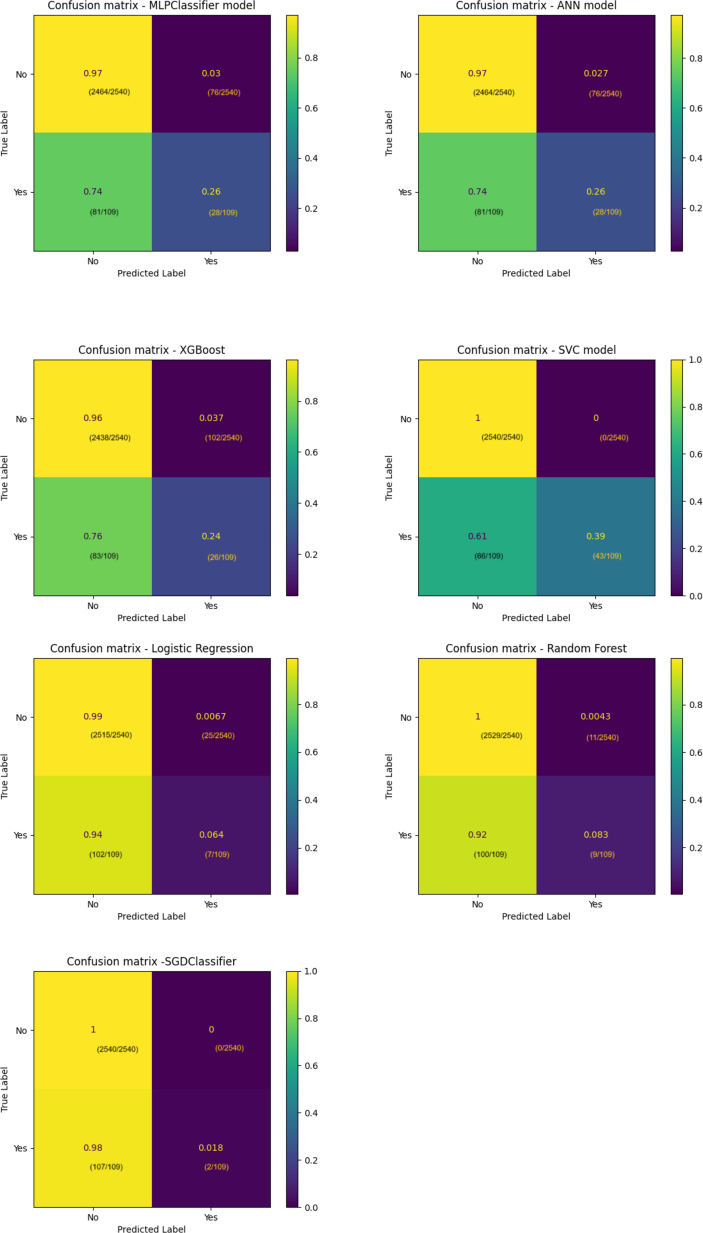
Confusion matrices for each classifier with a test dataset of 2649 patient records. ANN, artificial neural network; MLPClassifier, multilayer perceptron classifier; SGDClassifier, stochastic gradient descent classifier; SVC, support vector classifier; XGBoost, eXtreme gradient boosting.

Confusion matrices were created by selecting an arbitrary probability threshold of 0.4 for classifying VTE, which are presented in Figure 1. The confusion matrices highlight true positives (sensitivity—correctly predicting patients who develop thrombosis), true negatives (specificity—correctly predicting patients who do not develop thrombosis), false positives (incorrectly predicting thrombosis development), and false negatives (incorrectly predicting no thrombosis development).

**Figure 2:**
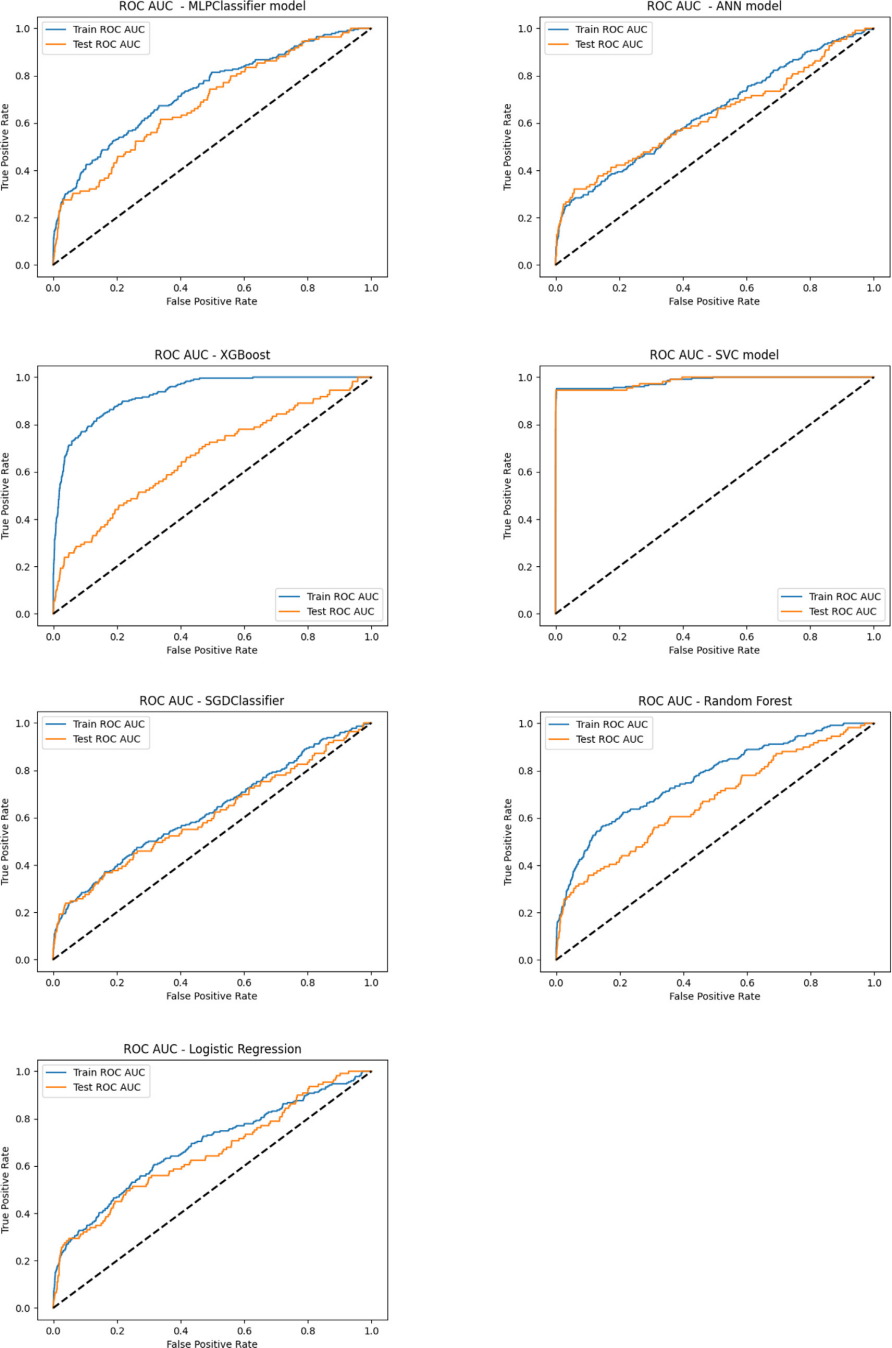
Receiver operating curve diagrams for each classifier. ANN, artificial neural network; MLPClassifier, multilayer perceptron classifier; ROC AUC, area under the receiver operating characteristic curve; SGDClassifier, stochastic gradient descent classifier; SVC, support vector classifier; XGBoost, eXtreme gradient boosting.

A summary of the results is provided in Tables 5 and 6 and Figures 1 and 2.

**Table 5:** Performance evaluation based on accuracy, F1 score, precision, and recall.

Model	Accuracy	Precision (macro/weighted)	Recall (macro/weighted)	F1 Score (macro/weighted)
Multilayer perceptron classifier	0.96	0.62/0.94	0.61/0.94	0.62/0.94
Artificial neural network	0.94	0.63/0.94	0.62/0.94	0.62/0.94
eXtreme gradient boosting	0.93	0.59/0.94	0.60/0.93	0.60/0.93
Support vector classifier	0.97	0.99/0.98	0.69/0.97	0.77/0.97
Stochastic gradient descent classifier	0.96	0.98/0.96	0.51/0.96	0.51/0.94
Random forest	0.96	0.71/0.94	0.54/0.96	0.56/0.94
Logistic regression	0.95	0.63/0.93	0.53/0.96	0.54/0.94

**Table 6:** Performance evaluation based on log loss and area and the curve score.

Model	Log loss (training data/test data)	ROC AUC (training data/test data)
Multilayer perceptron classifier	0.153/0.157	0.732/0.685
Artificial neural network	0.160/0.157	0.64/0.63
eXtreme gradient boosting	0.118/0.162	0.93/0.663
Support vector classifier	0.110/0.112	0.984/0.983
Stochastic gradient descent classifier	1.412/1.361	0.628/0.608
Random forest	0.155/0.157	0.767/0.669
Logistic regression	0.158/0.158	0.682/0.655

ROC AUC, area under the receiver operating characteristic curve.

After evaluating all key metrics including accuracy, precision, recall, F1 score, loss score, and ROC AUC score, we determined that the SVC model delivered the best overall performance in predicting development of VTE in COVID-19 patients, outperforming all other models.

## 4 Discussion

In this study, by using AI algorithms and standard statistical analysis, we identified demographic factors (increasing age, being female, white ethnicity), comorbidities (diabetes, liver disease, autoimmune disease), and laboratory features (raised hemoglobin, white cell count, D-dimer, lactate dehydrogenase, ferritin) and presence of MOF, as major contributory factors for developing VTE in hospitalized COVID-19 patients. Using these features, we developed and compared 7 AI models to predict the development of VTE in patients with COVID-19 and found that that the SVC model outperformed all other models, achieving an accuracy of 97%. Notably, it was 100% accurate in predicting the absence of thrombosis (true negatives) and surpassed other models in accurately predicting development of VTE (true positives). The SVC model also led in precision (0.98), recall (0.97), and F1 score (0.97), and recorded the lowest log-loss score (0.112 on the test dataset), reflecting better model convergence and an improved fit to the data. Additionally, it achieved the highest AUC score (0.983), a key metric for evaluating the model’s ability to distinguish between positive and negative cases. A higher AUC score highlights its superior discriminatory power. Following the SVC, both MLP Classifier and ANN models also demonstrated strong overall performance, consistently outperforming other models across key metrics, including accuracy, precision, recall, F1 score, log-loss score, and AUC score. For example, the study on “individual-level fatality prediction of COVID-19 patients using AI methods” reported accuracy and specificity just above 90% in its top-performing autoencoder model.

Our best-performing model (SVC) is an implementation of the Support Vector Machine algorithm, and it finds the hyperplane that best separates the data points into different classes. It achieved an accuracy of 97%, outperforming similar studies that developed deep-learning and machine-learning models on data related to COVID-19 patients. Since all patients included into the models received thromboprophylaxis with low molecular weight heparin, the prediction models are not affected by this variable.

The use of AI to predict the development of VTE in hospitalized patients has been studied prior to the COVID-19 pandemic. A study by Ryan et al. [5] used machine-learning to predict DVT among hospitalized patients. The study included a total of 99 237 patients and of these patients, 2378 experienced DVT during their hospital stay. It developed and validated a gradient gradient-boosted learning algorithm to predict a patient’s risk of developing DVT at 12- and 24-hour windows prior to onset, enhancing clinicians’ ability to identify and monitor high-risk patients. It used features such as cancer history, VTE history, and INR for building the machine-learning predictors obtained area under the receiver operating characteristics of 0.83 and 0.85 for DVT risk prediction on hospitalized patients at 12- and 24-hour windows, respectively. Similar to our study, DVT prediction in COVID-19 patients developed by Zhang et al. [6], which included a smaller number of patients (only 424) also incorporated features such as age, sex, BMI, PT, INR, CRP, TNF, IFN with model achieving an accuracy of 91.02% and a sensitivity of 91.07%.

The main strengths of this study are the use of a large number of patients to develop the model, data accuracy, as the data were collected by clinicians with appropriate clinical knowledge, and representation of the overall patient population in the UK, as the data were collected from 26 NHS Trusts in England, Wales, and Scotland during a specific period. In addition, the study used 7 models to find the best predictive model.

As the data used for the development of AI models were obtained from patients with COVID-19 admitted to hospitals in early 2020, during the peak of the pandemic when thrombosis and severe illness due to COVID-19 were much higher, these models will no longer be useful in predicting VTE in patients with COVID-19 in clinical practice at present as the disease is milder and the risk of VTE much lower. Although our models do not help in the clinical practice for COVID-19 at present, the methodology of this study (ie, data cleaning, AI-driven data imputation, feature selection, model building, cross-validation, hyperparameter tuning, and testing) can be used as a framework for the future disease prediction models. This cross-disease applicability is possible due to shared data patterns, underlying biological mechanisms, shared risk factors for disease outcomes (eg, some of the patient factors associated with increased risk VTE) and adaptability of the AI algorithms. Furthermore, the lack of validation using an external dataset is a limitation of the study as there is a possibility that our best-performing SVC model may be overfitted to the data.

In conclusion, we developed and compared 7 AI models for predicting the development of VTE in hospitalized COVID-19 patients using patient demographics, comorbidities, and on-admission laboratory data. Our best-performing model achieved an accuracy of 97%, outperforming similar studies that developed deep-learning and machine-learning models on data related to COVID-19 patients. In addition to the outstanding performance of the developed models, the unique contribution of this study is the specific focus on predicting VTE in COVID-19 patients, a clinical outcome not addressed by other AI-driven COVID-19 studies in the published literature, particularly using a dataset of this size. Although the models cannot be used to predict VTE in patients admitted to hospitals with COVID-19 in current clinical practice due to the milder form of the disease and lower risk of VTE, techniques used in this study can be repurposed for other clinical conditions.

## Appendix

**CA-COVID19 Study Collaborators**

Aneurin Bevan University Health Board, Wales, United Kingdom: Amanda Dell, Angela Hall, Anna Roynon, Anne Heron, Cheri Price, Claire Price, Clare Westacott, Debra Barnett, Gail Marshall, Gemma Hodkinson, Georgia Mallison, Grace Okoro, Joshua Gwatkin, Kirstin Davies, Lucy Shipp, Maxine Nash, Rhian Hughes, Rina Mardania, Sarah Lewis Sean Cutler; Aberdeen Royal Infirmary, Aberdeen, United Kingdom: Caroline Allan; Barts Health NHS Trust, London, United Kingdom: Atiqa Miah, Dide Okaygun, Dan Hart, Faith Dzumbunu, James Leveson, Karen Torre, Louise Taylor, Priyanka Raheja, Sara Mamsa, Tasnima Ferdousi; Buckinghamshire Healthcare NHS Trust, Buckinghamshire, United Kingdom: Angharad Everden, Alice Ngumo, Doaa Ahmed, Efstathia Venizelou, James Herdman, Janice Carpenter, Konrad Bartkiewicz, Rebecca Cash Renu Riat; Cardiff and Vale University Health Board, Cardiff, United Kingdom: Abigail Downing, Ana Guerrero, Astrid Etherington, Chapa Gamage, Dilupa Gunasekara, Lee Morris, Raza Alikhan, Rebecca Cloudsdale, Samya Obaji, Stuart Cunningham, Sylvain Ndjombo; County Durham and Darlington NHS Foundation Trust, Darlington, United Kingdom: Amanda Cowton, Ami Wilkinson, Andrea Kay, Anne Sebakungu, Anne Thomson, Clare Brady, Dawn Egginton, Ellen Brown, Enid Wright, Gill Rogers, Hannah Plaschkes, Jacqui Jennings, Julie O’Brien, Julie Temple, Kathryn Potts, Kimberly Stamp, Kelly Postlethwaite, Louise Duncan, Margaret Randall, Mark Birt, Melanie Kent, Philip Mounter, Shelly Wood, Nicola Hewitson, Noreen Kingston, Susan Wadd, Sarah McAuliffe, Stefanie Hobson, Susan Riley, Suzanne Naylor, Vicki Atkinson; Cwm Taf Morgannwg University Health Board, Wales, United Kingdom: Alysha Hancock, Bethan Deacon, Carla Pothecary, Caroline Hamilton, Ceri Lynch, Cerys Evenden, Deborah Jones, Ellie Davies, Felicity Page, Gareth Kennard-Holden, Gavin John, Joanne Pugh, Joelle Pike, Justyna Mikusek, Kevin Agravante, Kia Hancock, Lauren Geen, Meryl Rees, Natalie Stroud; Gateshead Health NHS Foundation Trust, Gateshead, United Kingdom: Amanda Grahamslaw, Amanda Sanderson, Beverley McClelland, Caitlin Barry, Elaine Siddle, Lorraine Pearce, Lucy Blackwell, Maria Bokhari, Maureen Armstrong, Wendy Stoker, Wendy McCormick; Guy’s and St Thomas’ NHS Foundation Trust, London, United Kingdom: Caterina Vlachou, Ben Garfield, Mihaela Gaspar, Maurizio Passariello, Paolo Bianchi, Stephane Ledot; Hampshire Hospitals NHS Foundation Trust, Basingstoke, United Kingdom: Aileen Madlin, Kerrianne Everard, Khushboo Panwar, Natasha Beacher, Niamh Cole, Sarah Mangles, Tamara Everington, Udaya Reddy; Imperial College Healthcare NHS Trust, London, United Kingdom: Alka Shah, Anna Weatherill, Anthi Maropoulou, Bhagya Herath, Billy Hopkins, Camelia Vladescu, Caroline Ward, Christina Crossette-Thambiah Donna Copeland, Emily Pickford, Gaurika Kapoor, Isabella Lo, John Kilner, Keith Boland, Melanie Almonte, Neil Simpson, Niamh Bohnacker, Omolade Awomolo, Roochi Trikha, Samina Hussain, Serah Duro, Sophie Kathirgamanathan, Yasmine Needham, Yee Hui, Zainab Alashe; King’s College Hospital NHS Foundation Trust, London, United Kingdom: Adrienne Abioye, Aileen Miranda, Christina Obiorah, Cynthia Dzienyo, Hasina Mangal, Hernan Zorraquino, Lara N Roberts, Mariusz Racz, Maclaine Hipolito Johnson, Rachel Ryan, Tamara Swales, Tatiana Taran, Zoe Renshaw; Newcastle Hospitals NHS Foundation Trust, Newcastle, United Kingdom: Alexander Langridge, Benjamin Evans, Callum Weller, Claire Judd, Douglas Jerry, Euan Haynes, Fatima Jamil, Ian McVittie, John Hanley, Julie Parker, Kayleigh Smith, Keir Pickard, Laura Kennedy, Meghan Acres, Mikaela Wiltshire, Nitha Ramachandran, Paul McAlinden, Paula Glancy, Smeera Nair, Tarek Almugassabi, Thomas Jarvis; NHS Grampian, Scotland, United Kingdom: Amanda Coutts, Andrew Laurie, Deborah Owen, Ian Scott, Jamie Cooper, Leia Kane, Lucy Sim, Mahmoud Abdelrahman, Victoria Poulton; Norfolk and Norwich University Hospitals NHS Foundation Trust, Norfolk, United Kingdom: Jessica Griffin, Ria Markwell, Suzanne Docherty; North Cumbria Integrated Care NHS Foundation Trust, Cumbria, United Kingdom: Alexander Brown, Barbara Cooper, Beverley Wilkinson, Diane Armstrong, Grace Fryer, Jane Gregory, Katherine Davidson, Melanie Clapham, Nicci Kelsall, Patricia Nicholls, Rachel Hardy, Roderick Oakes, Rosemary Harper, Sara Abdelhamid, Theresa Cooper, Una Poultney, Zoe Saunders; North Tees and Hartlepool NHS Foundation Trust, Durham, United Kingdom: Alex Ramshaw, Alison Chilvers, Barbara Jean Campbell, Carol Adams, Claire Riley, Deborah Wilson, Helen Wardle, Jill Deane, Jill Skelton, Julie Quigley, Leigh Pollard, Liz Baker, Lynda Poole, Maria Weetman, Michele Clark, Nini Aung, Rachel Taylor, Sarah Rowling, Sarah Purvis, Vicky Collins: Northumbria Healthcare NHS Foundation Trust, Newcastle Upon Tyne, United Kingdom: Amy Shenfine, Catherine Ashbrook-Raby, Charlotte Bomken, Claire Walker, Faye Cartner, Helen Campbell, Jane Luke, Jessica Reynolds, Mari Kilner, Laura Winder, Linda Patterson, Lisa Gallagher, Nicola McLarty, Sandra Robinson, Steve Dodds, Toni Hall, Victoria Wright; Oxford University Hospitals NHS Foundation Trust: Agnes Eordogh, Alexandros Rampotas, Anna Maria Sanigorska, Christopher Deane, Kristine Santos, Olivia Lecocq, Rochelle Lay, Simon Fletcher,not Susie Shapiro; Royal Free London NHS Foundation Trust, London, United Kingdom: Anna Tarnakina, Aniqa Tasnim, Anja Drebes, Cecilia Garcia, Elsa Aradom, Mariarita Peralta, Michaella Tomlin, Pratima Chowdary, Ramona Georgescu, Suluma Mohamed, Upuli Dissanayake; Royal Liverpool and Broadgreen University Hospitals NHS Trust, Liverpool, United Kingdom: Carol Powell, James Doolan, Jessica Kenworthy, Joanne Bell, Lewis Jones, Mikiko Wilkinson, Rebecca Shaw, Ryan Robinson, Saman Mukhtar, Shane D'Souza, Tina Dutt, Tracy Stocks; Royal Papworth Hospital NHS Foundation Trust, Cambridge, United Kingdom: Joshua Wade, Lenka Cagova, Maksym Kovzel, Rachel Jooste; Sheffield Teaching Hospitals NHS Foundation Trust, Sheffield, United Kingdom: Alison Delaney, Claire Mapplebeck; South Tees NHS Foundation Trust, Middlesbrough, United Kingdom: Alycon Walker, Andrea Watson, Andrew Vaux, Asia Sawar, Carol Hannaway, Charlotte Jacobs, Claire Elliot, Claire Elliott, Craig Mower, Daiana Ferro, Emanuela Mahmoud, Gill Laidlaw, Julie Potts, Keith Harland, Laura Munglani, Lauren Fall, Leanne Murray, Lesley Harris, Lisa Wayman, Lisa Westwood, Louisa Watson, Lynne Naylor, Matthew Siddaway, Paula Robson, Rita Mohan, Sarah Essex, Sara Griffiths, Steven Liggett; University Hospital Southampton NHS Foundation Trust, Southampton, United Kingdom: Andreia Valente, Rashid Kazmi, Ruth Kirby, Sarah Bowmer, Yanli Li; University Hospitals Birmingham NHS Foundation Trust, Birmingham, United Kingdom: Alice Longe, Amy Bamford, Anand Lokare, Andrew McDarby, Aneta Drozd, Cathy Stretton, Catia Mulvihill, Charlotte Ferris, Christopher McGhee, Claire McNeill, Colin Bergin. Daniella Lynch, Fionnuala Lenehan, Gerry Gilleran, Gillian Lowe, Graham McIlroy, Helen Jenner, Helen Shackleford, Isma Younis, Jaspret Gill, Jimmy Musngi, Joanne Dasgin, Joanne Gresty, Joseph Nyaboko, Juneka Begum, Katerine Festejo, Katherine Lucas, Katie Price, Khushpreet Bhandal, Kristina Gallagher, Kyriaki Tsakiridou, Lauren Cooper, Louise Wood, Lulu Amutike, Marie Thomas, Marwan Kwok, Melanie Kelly, Michelle Bates, Nafeesah Ahmad Haider, Nicholas Adams, Oliver Topping, Rachel Smith, Rani Maria Joseph, Salma Kadiri, Samantha Caddick, Samuel Harrison, Shereef Elmoamly, Stavroula Chante, Sumaiyyah Gauhar, Syed Ashraf, Tabinda Kharodia, Zhane Peterkin; University Hospitals of Leicester NHS Trust, Leicester, United Kingdom: Isgro Graziella, Hakeem Yusuff; University Hospitals of North Midlands NHS Trust: David Sutton, Ian Massey, Jade Di-Silvestro, Joanne Hiden, Mia Johnson, Richard Buka; University Hospitals Plymouth NHS Trust, Plymouth, United Kingdom: Claire Lentaigne, Jackie Wooding, Nicola Crosbie; Whittington Health NHS Trust: Ana Alvaro, Emma Drasar, Elen Roblin, Georgina Santiapillai, Kathryn Simpson, Kayleigh Gilbert, Yanrong Jiang, Zara Sayar, Zehraa Al-Khafaji

## Figures and Tables

**Supplementary Figure S1:**